# Norwegian Shelters for Victims of Domestic Violence in the COVID-19 Pandemic – Navigating the New Normal

**DOI:** 10.1007/s10896-021-00273-6

**Published:** 2021-04-08

**Authors:** Solveig Bergman, Margunn Bjørnholt, Hannah Helseth

**Affiliations:** Norwegian Centre for Violence and Traumatic Stress Studies, Oslo, Norway

**Keywords:** Domestic violence, Shelters for victims of domestic violence, Domestic violence and COVID-19, Coronavirus and domestic violence, Social services during COVID-19, Domestic violence and crisis, Violence and pandemic, Coercive control

## Abstract

This study elucidates the responses of shelters and their adaptations to the COVID-19 pandemic, and the effects on their services to victims of violence, as well as how shelter managers assess the situation for victims, including changes in the rates and character of the violence observed by the shelters. A web-based survey was distributed twice to all Norwegian shelters (*N* = 46): first during the lockdown in spring 2020 and second during the relaxation of infection control measures in summer 2020. The shelters in Norway remained open during the COVID-19 pandemic. The majority saw a reduction in the number of requests during the lockdown, while the rates returned to normal when the strictest infection control measures were lifted. They expressed concern about the decline in requests during the lockdown as well as the well-being of some groups, such as victims from ethnic minority backgrounds, children, and victims with additional challenges. A majority of the shelters did not report changes in the content of the requests. Nevertheless, a third of them had observed instances of the virus and/or infection control measures being used by perpetrators as part of the violence and coercive control strategies. The shelters in Norway, as an integrated part of the welfare state, in general seem to have met the needs of their clients during the pandemic. Yet, the study revealed important inequalities and deficiencies in access to services for some groups, and in the general support and recognition by authorities of the shelters.

## Introduction

Early in the COVID-19 pandemic, reports both in media and by international organizations emerged of an increase in domestic violence or risk of such violence in the wake of infection control measures (Graham-Harrison et al., 2020; UN Women, 2020; World Health Organization, 2020). However, the picture seems to be more mixed. While some countries seemed to observe an increase in reports of domestic violence during the lockdown^1^ of society, others saw a decline in the reporting of such violence (Boserup et al., 2020; Brooks-Hay et al., 2020; EURACTIV Network, 2020).

After the first reports of a “spike” or “surge” in domestic violence, warning voices were raised against unilaterally focusing on trends and changes in rates of violence. Williamson et al. (2020) argue that this media narrative implicitly constructs a causal relation between the pandemic and domestic violence, and further that this narrative prioritizes a focus on physical one-off incidents of domestic violence, while ignoring coercive control rooted in gendered patterns of domination. This critique is corroborated by research that suggests that the pandemic has exacerbated existing structural concerns for victims of domestic violence and sexual assault (Wood, Baumler, et al., 2020a), and has led to an increase in the severity of violence against women and children (Ertan et al., 2020; Peterman & O’Donnell, 2020).

Dartnall et al. (2020) similarly argue that we need to shift the emphasis to include questions such as how the pandemic influences the situation for those who live with domestic violence and whether victims have been able to access services:

The important questions to ask during this pandemic are not whether the violence has increased or decreased (which we cannot answer). We should rather be asking about the impact of the virus and social distancing measures on women and children, and whether they’ve been able to access services. (…) This question is best answered by those working with women and children on the frontline.

We agree that there is a need to move beyond the question of a rise or decline in domestic violence, as well as to move beyond simplistic assumptions that see the pandemic as the direct cause of an increase of such violence. Hence, the main concern in this article is to highlight how the pandemic has impacted the support services for victims of domestic violence and how the frontline workers experience and assess the situation for victims of domestic violence during the pandemic.

Comparative research shows that the overall approaches and responses to domestic violence, as well as the models of societal intervention, vary across countries (Hagemann-White, 2010). In Norway, this intervention is largely constructed around the protection and support of victims, although control aspects and criminal prosecution of perpetrators are also of increasing importance (Bakketeig et al., 2019). The shelters are thus key informants on domestic violence and provide a privileged vantage point for obtaining knowledge about such violence in Norway. Moreover, the shelter leaders and employees are knowledgeable and competent when it comes to handling domestic violence and carrying out risk assessment. Although the question of an increase or decrease in domestic violence is not the primary concern in this article, we consider shelter leaders and employees as being well positioned to observe any changes in the frequency and character of violence in the populations that use their services that are relevant for the services they provide.

In the following section, we give an overview of the development of shelter services in Norway and the role of the shelters in combatting domestic violence and providing support for their victims.

## Shelters for Victims of Domestic Violence in Norway

For several decades, women’s shelters have been important actors in efforts to prevent and combat domestic violence in Norway, and they have had a pivotal role in the provision of assistance and services for victims of domestic violence. As elsewhere in the Western countries, the Norwegian shelters established by activists in the feminist movement in the 1970s were one of the first forms of intervention in domestic violence (Jonassen, 2005; Tierney, 1982). Over the years, the shelter movements across the world have played an important role in practicing feminist ideals by focusing on women and their experiences of domestic violence, and by raising the topic to its current level of awareness on the public and political agenda (Kelly et al., 2011).

The shelters offer low-threshold temporary accommodation for victims of domestic violence, as well as support and assistance for adults and children not living at the shelter, a group called “day-users.” Staying at a shelter is free of charge and does not require referrals. The shelters were initially staffed by volunteers and relied at least partly on funding from charities and other private donors. Yet, from the very beginning, the shelters also received public funding and support from the authorities (Skjørten et al., 2019).

Over the last few decades, domestic violence has gained increasing recognition in Norwegian society as a social problem, demanding the attention and focus of both the authorities and society as a whole. In Norway, the collaboration between the shelter movement, policy, and research has been a driving force behind improving the shelter services and bringing the issue up in the political arena (Skjørten et al., 2019).

In 2010, a new act relating to municipal shelter services entered into force in Norway. It imposed responsibility on all municipalities to offer their inhabitants services according to the requirements of the act. The purpose of the Shelter Act is to ensure the provision of good, comprehensive shelter services to women, men, and children who are subjected to domestic violence or threats of such violence (Bakketeig et al., 2014). In recent years, the focus on children in shelters has increased in both Norway and other Nordic countries (Selvik & Øverlien, 2014). Municipalities are obliged to offer adequate and appropriate accommodation at a shelter, as well as daytime services, such as advice, support, and guidance. Today, about half of the shelters in Norway are run and owned by municipalities or are inter-municipal entities, and the rest are owned by private foundations or NGOs (Bliksvær et al., 2019). The large majority of the staff at Norwegian shelters are employed full- or part-time, and the number of volunteers is very small today. Almost 60% of the staff have college- or university-level education (Shelter statistics, 2019).

As of 2020, there were 46 shelters distributed throughout Norway, a country of 5.4 million inhabitants. Because the provision of shelter services is mandated by law, these services are fully funded by the local authorities. According to a cross-national comparison, Norway is one of the very few countries in Europe that meet the Istanbul Convention^2^ requirements for shelter provision (Wave Country Report, 2019). However, an evaluation of the implementation of the Shelter Act (Bakketeig et al., 2014; see also Bliksvær et al., 2019) indicates that several shelters continue to be hindered by limited budgets, and the legal obligation to provide shelter services of equal quality and accessibility throughout the country has not as yet been realized. Due to structural reform after the “municipalization” of shelter services, the number of shelters has been reduced from 55 to the current 46. In some parts of the country, large distances are an additional impediment to accessing shelters and other support services (Shadow report, 2020).

Moreover, the deficiencies in the Norwegian State’s compliance with its human rights obligations to the indigenous Sámi people have to be underlined here (Norwegian National Human Rights Institute, 2018). In 2019, the Sámi Shelter in Karasjok, the only one in the country with expertise about the Sámi people, was closed due to financial problems in the municipality. Yet, research in Norway shows that the Sámi, particularly women and children, are exposed to more violence and abuse than non-Sámis living in the same region (Eriksen et al., 2015).

According to the Shelter Act, the authorities must support the shelters and other municipal services, as well as NGOs, so that these can provide comprehensive assistance for the clients after they have left the shelter. Research and evaluations show, however, that these stipulations have not been met, and there is significant variation within the country in the extent of the provision of follow-up measures by the municipal authorities (Bakketeig et al., 2014; Bliksvær et al., 2019).

Each year statistics from the shelters in Norway are collected, analyzed, and published. The latest shelter statistics (2019) show that 1800 adult persons (92% of them women) and 1450 children were residents in shelters in Norway, and over 2600 persons (91% of them women) used the daytime services of the shelters; 63% of all residents and 47% of nonresident clients had a non-Norwegian ethnic background (Shelter statistics, 2019). Thus, clients with migrant backgrounds are overrepresented compared to their proportion of the population in general. Many women and children from ethnic minority families who flee from domestic violence experience multiple constraints in their lives (Kiamanesh & Hauge, 2019).

## Aims, Data, and Method

This study has two main objectives: firstly, to examine the responses of shelters and their adaptations to the pandemic and its effects on their services to victims of domestic violence; secondly, to study how shelters, as key informants on the frontline, assess the situation for victims, including changes in rates and the character of domestic violence as observed by the shelters during the pandemic.

A web-based survey was distributed to the leaders of all 46 Norwegian shelters for victims of domestic violence during the lockdown period in spring 2020 (T1) and after the gradual reopening of society in early summer 2020 (T2). The survey contained four sections covering (1) institutional adaptations or changes due to infection-control measures and cooperation with other services (e.g., *Have the services your shelter offers changed in any way since the strict infection control measures were introduced? Have you experienced changes in the staff situation? How has the pandemic affected the cooperation with other service providers and local/national agencies and authorities?)* (2) The shelters’ suggestions for improvement (e.g., *What measures need to be undertaken to improve services during the pandemic?* (3) Shelters’ assessment of possible changes in the number and character of requests from victims during the pandemic (e.g., *Have you experienced changes in the rates of requests from users? Or in the character/contents of the requests? Have you found that the users report that the virus/infection control measures are used as part of the control or the violence they are subjected to?*) (4) The shelters’ concern about specific groups of victims (*Are you especially concerned about certain groups of victims? If so, what groups are you referring to?*).

The shelters were given questions with fixed responses, with options such as yes/no, or they could choose between relevant alternatives from a list, for instance different types of measures in response to the pandemic, cooperation with particular other services, or different forms of corona related violence. We included qualitative follow-up questions to most questions in the survey in order to give the respondents the opportunity to elaborate on their fixed answers. The quantitative data were summarized using descriptive methods (absolute numbers and percentages), and the qualitative data were used to illustrate the findings.

The initial survey was carried out in April, and the follow-up survey, with somewhat revised questions, in June/July. The response rate was 100% (*n* = 46) and 80% (*n* = 37), respectively. The time period for filling out the second survey was in the midst of the summer vacation period in Norway, which may have influenced the response rate.

The main results from both the initial and the follow-up surveys will be presented in the next sections. First, we discuss the institutional changes introduced at the shelters due to the pandemic, their cooperation with other services and agencies, and the shelters’ suggestions to improve their services. The second part describes the groups about which the shelters expressed particular concerns. Finally, the third part deals with changes in the requests and contacts made by victims to the shelters and the possible changes in the character of domestic violence in this period.

## Institutional Changes and Challenges

In Norway, the shelters remained open during the lockdown period, but the majority reduced or adapted their services in line with the infection control measures; in particular, this affected counseling and advice to nonresident clients, group activities, and home visits (Table 1). Such services had to be carried out primarily by telephone or via digital platforms. Nine out of 10 shelters reported that they had to establish new communications channels with their users or introduce other new routines for meeting them. Particularly at T1, several shelters (60%) were also obliged to make changes for the resident clients, including in the admission requirements for new residents. For example, some shelters reported that they had to try to find alternative solutions for clients together with other services, the police, or the client’s own networks, and other shelters pointed out that they were only able to admit clients who were in acute danger.

**Table 1:** Changes in shelter services during the pandemic (several response alternatives were possible)

	During the lockdown period (n/46)	After the reopening of society (n/37)
Limitations of activities and group offer	33	13
Changes in services for ambulatory clients	32	8
Reduction of home visits	25	9
Changes in services for resident clients	14	7
Reduction of staff	14	5
No changes in services	2	13

Nearly 70% of the shelters reported staff reductions, primarily due to employees being in quarantine, isolation, or on sickness leave, but also because of staff members who had to take care leave because of closed daycare centers and schools. On March 23, 11 days after lockdown, shelter employees were defined as a “workforce in critical societal functions”—that is, their children had to be provided access to childcare and schools despite the lockdown, and shelter employees were not to be transferred to other work tasks in the municipalities (Norwegian Directorate for Children, Youth, and Family Affairs, 2020). Despite this new rule, a third of the shelters reported experiencing staff shortages at T1 (Table 1), as this regulation concerned only families where *both parents* were defined as workforce in a critical societal function.

After the gradual reopening of society from May 2020 onwards, the shelters were able to relax the restrictions implemented during the lockdown phase. Yet, even at this point of time, a third of the shelters reported having to restrict activities and group-based services to their clients, and at T2 two thirds of the shelters reported that they still did not operate in the same way that they did prior to the pandemic.

As a mandatory nationwide service, the shelters are part of the local welfare services, and they operate in close cooperation with other services. Both the initial survey (T1) and the follow-up survey (T2) showed that the shelters received support from the municipalities due to the pandemic, more than half of them at T1, and above 20% at T2.

Most shelters (74–100% depending on the services) claimed that there had been no change during the pandemic in their cooperation with other services offered by municipalities, state authorities, or private institutions compared to the normal situation prior to the pandemic. However, this need not necessarily mean that this cooperation was functioning in an optimal way. When asked what the shelters considered to be the most important measures or courses of action for being able to deliver good services during the pandemic, over 80% of the shelters emphasized that cooperation with local authorities and services was the most important activity. Almost the same number considered good information from the national authorities about COVID-19 and protection rules to be key in this respect.

At both T1 and T2, around 70% of the shelters stated that societal recognition of their role and competence during the pandemic was crucial. In the commentary field, some of the shelters elaborated on the issue of recognition. One shelter wrote: *“We hope that recognition of our work would have financial repercussions in the future (…). For our part, we do this because we want to give support to these women, men and children, not to get recognition.”* Several shelters emphasized that their staff had made considerable efforts to continue to support the victims while simultaneously having to follow the infection control rules. Yet, as another shelter points out, there is considerable variation with regard to financial resources available to the shelters, which also affects their ability to provide equitable services throughout the country. A common concern for the future amongst the shelters was expressed as follows:

That we have been able to deliver good services during the pandemic is thanks to the willingness of our staff to act, to set their own needs aside in order to focus on how to help victims of violence in the best possible way. To gain recognition and to be treated as having equal value is important for us in order to be able to continue to deliver high-quality services. When the authorities regards us as important actors this contributes to feelings of such satisfaction. Many of our staff are exhausted when they go on vacation in the summer. The pandemic will most probably continue, and we will have to stick to many of the measures.

Close to 60% of the shelters expressed concerns over their financial situation and resources at both T1 and T2. This comment is illustrative of these concerns:

The important thing is the external conditions for shelters in general, in terms of staffing, competence requirements, security. The situation with COVID-19 is just one of many challenges. Had the external conditions of the centers and their clients generally been better, the COVID-19 situation would have been easier to handle.

Several shelters elaborated on their financial worries due to extra costs they had to bear during the pandemic. One wrote simply: *“The most important thing is financial compensation for additional expenses.”* Along with the financial and staffing situation, unsuitable premises were also emphasized. The unsuitability of premises became particularly apparent during the pandemic: *“Suitable premises are also important. Old, outdated premises that are unsuitable for modern operations and (they) compromise infection control considerations. One of the consequences is that not all rooms in the center can be used.”*

Despite the need to adapt and adjust their services during the pandemic, and problems such as staff shortages, on the whole the shelters have continued to function throughout the pandemic. However, the COVID-19 crisis has revealed certain weaknesses and deficiencies affecting the shelters, such as the underfunding and the insufficient external conditions and societal recognition of the role of the shelters. These issues have long been a point of concern for the shelters, and it was one of the critiques raised in the shadow report from Norwegian NGOs to GREVIO, the group of experts appointed by the Council of Europe to monitor the implementation of the Istanbul Convention (Shadow report, 2020).

A further challenge that was emphasized by the shelters was their concern particularly for certain groups of victims of domestic violence during the pandemic, as will be discussed in the following section.

## Concerns for Particular Groups

Several of the shelters expressed particular concern that some of the most vulnerable groups, such as ethnic minorities and children, did not get the services and protection they needed, in view of the fact that the infection control measures led to a general reduction in social contacts and also in the provision of several other services for victims of violence. During the lockdown period, 84% (*n* = 36) of the shelters were particularly worried about the situation of children (cf. Øverlien, 2020). One shelter described the situation for children staying at the shelter as follows: *“It has been really demanding with all the children who have not gone to school, but stayed at the shelter 24/7 with no alternative activities. Digital schooling and support for parents in crisis. We need more resources for those kids.”*

During the reopening phase, when childcare centers and schools had opened their doors again, this concern was reduced. Yet, even at this point of time, 57% (*n* = 21) of the shelters expressed particular concern for the children.

About half of the shelters (*n* = 22) were particularly worried about victims with a migrant or ethnic minority background at T1, and 43% (*n* = 16) were worried about this group during the reopening phase. At both T1 and T2, 57% of the shelters (*n* = 23/21) were particularly worried about other vulnerable groups, such as pregnant women and victims with substance abuse or other additional problems.

Due to the lockdown, almost all of the ordinary meeting places for refugees and migrants closed, such as language courses or training and local initiatives. This made it more difficult to identify cases of violence and to find ways of dealing with it. One shelter manager expressed her concern in the following way:

Most services, schools, daycare centers, and language and training courses for refugees and other persons with minority backgrounds are now closed. Victims of domestic violence from ethnic minority families are often picked up by such institutions. Even the services for asylum seekers and refugees are closed, and labor and welfare offices only offer services over the phone. We fear that these groups will not get the help that is so necessary for them, and that includes the support we at the shelters could give to them.

The reopening of society may have led to other services providing referrals and advice to victims of domestic violence. One shelter commented: *“The Child Welfare Service and the police are making more contact now after the reopening. We also have significantly more families with children now than earlier during the pandemic.”*

The reopening of society, including public services, facilitated the provision of support to victims of domestic violence. Yet, as the comment quoted below indicates, the situation remained difficult for several groups:

When it comes to the children, it is a short time since the schools reopened, and now it is summer vacation—which means that the arenas for adult contact outside of the family are limited. Victims of violence with an ethnic minority background have less access to information, and therefore they are more uncertain of the situation than the majority of the population. Many of the services for people with substance abuse and/or mental health problems have been limited for a long period, and we have holiday cancellations as well, which make them more vulnerable.

This shelter’s concern sums up many of the comments at both T1 and T2 on how the restrictions can affect particular groups of victims. Furthermore, the retrenchment or scaling back of other services and activities in society may negatively affect victims of violence in various ways and limit their ability to access a range of services and support, including shelter services.

The worries expressed by the shelters mirror recent studies in Norway about the impact of the pandemic and restrictions on minorities. Diaz et al. (2020) argue that information about the pandemic aimed at migrant groups was not part of the general pandemic response from the beginning in Norway, and the information was poorly adapted to their needs. Another important study was carried out by the MIRA Center—a Norwegian NGO for women with ethnic minority backgrounds. Their report emphasizes that many minority women felt that they had been left alone and isolated during the pandemic and that many migrant women were unaware that social services, including the shelters, remained open during the lockdown period (MIRA Center, 2020). Further, although information on public websites about services for victims of violence has improved somewhat during the pandemic, it is mainly in Norwegian. This shortcoming also affects the national domestic violence hotline, where the English translation of the site is tucked away at the very end of the Norwegian text and information in other languages is not provided.

Whether the lack of information in foreign languages partly explains the reduction in the number of victims who contacted the shelters in the early phase of the pandemic, we cannot say. But it might be part of the shelter staff’s worries about the ominous silence during the lockdown. This study indicates that the pandemic and the restrictions may exacerbate existing inequalities in society, thus worsening the situation, particularly for those who are already in troubled and marginalized situations.

A study focusing on another important service in Norway, the Family Welfare Service, a public agency that provides mandatory mediation for parents seeking divorce, and counseling for couples and families, concluded that for families with moderate problems, the lockdown led to an improvement of family relations, while families with less financial resources and with higher conflict levels experienced a substantial increase in problems (Øverli & Gundersen, 2020).

Furthermore, a study that was carried out among Norwegian 12- to 16-year-olds in June 2020, a short time after schools reopened after two months of lockdown, shows that teenagers in vulnerable life situations were particularly affected by the corona crisis (Augusti et al., under review). They reported a greater incidence of violence and sexual abuse during the lockdown period than did other pupils. Girls were more exposed to almost all forms of violence and abuse than boys, and the gender difference was bigger and more consistent compared to the situation one year before the outbreak of the pandemic. Internet-based violence was particularly frequent in the lockdown period (Augusti et al., under review).

The studies referred to above support the observations of the shelters, and draw attention to underlying structural inequalities in Norwegian society based on, for example, ethnicity and class, and how these became acute and were exacerbated during the pandemic, something we discuss further in the concluding section of this article.

The next section will present the findings concerning the shelters’ judgment of the frequency and the character of the violence experienced by their clients, particularly the issue of possible coronavirus-specific forms of violence and control.

## Changes in the Number of Requests and Forms of Violence Observed by the Shelters

As shown in Table 2, more than half of the shelters (*n* = 26) reported that there had been a reduction in the number of requests from victims during the lockdown period, while a small minority (16%, *n* = 7) reported an increase. Somewhat less than a third (*n* = 13) of the shelters reported that they received the same number of requests as before.

**Table 2:** Change in the number of requests from victims of domestic violence

Increase/decrease in the number of requests	During the lockdown, compared to before the pandemic (n/46)	After the reopening, compared to the lockdown	
		Resident clients (n/29)	Ambulatory clients (n/31)
Decrease	26	6	3
No changes	13	11	9
Increase	7	12	19

In the period following the reopening of society, the shelters were asked whether they had experienced changes in the number of requests compared with the lockdown phase (Table 2). The shelters have two groups of clients: ambulatory clients who are offered counseling and help, and resident clients who also receive temporary accommodation at the shelter. With regard to ambulatory clients, 59% (*n* = 19) of the shelters that answered this question reported that they had more requests during the reopening period than during the lockdown, and 28% (*n* = 9) reported that the situation was the same. Only three shelters reported having fewer inquiries after the reopening than during the lockdown period. For resident clients, 35% (*n* = 12) of the shelters reported that there are more inquiries now, 32% (*n* = 11) of the shelters said it was almost the same as during the lockdown, and 17% (*n* = 6) reported that they had fewer requests in the reopening period than during the lockdown period.

Some of the shelters added free text commentaries, offering more detail on the reported increase or decrease in requests. In the T1 survey, one shelter wrote: *“We have very few inquiries. In comparison, at the same time last year we had a completely full house and had to find other offers for 16 families due to lack of capacity. Now we have only two of eight rooms in use.”*

International media reports have drawn attention to the issue of how the coronavirus and infection control measures were used by perpetrators of violence as a new means to pressure, threaten, or control their partner (Gearin & Knight, 2020; Godin, 2020). Inspired by these reports, the survey addressed possible changes in the character of violence. Both at T1 and T2, a large majority of the shelters reported that they had not observed any changes in the forms or severity of violence. The fact that the shelters had not observed any changes in the character of violence is interesting and contradicts media stories of an increase in severe and life-threatening violence. However, given the general reduction in requests, and the relatively short time span, this finding should be interpreted with caution.

We also asked more specifically whether the shelters had reason to believe that the COVID-19 crisis or the infection control measures had been used directly by perpetrators as part of the control or violence strategies to which their victims were exposed. At both T1 and T2, a third of the shelters reported that they had observed examples of the virus or the infection control measures being used by perpetrators as part of their efforts to exercise domestic violence/coercive control.

Given the short period of time since the outbreak of the pandemic and the lockdown, and the reduction in the number of requests in the period, we think this is a notable finding, even though the majority (67–70%) of the shelters had not noticed any indication of the infection control measures or the COVID-19 crisis being used directly in this way. These findings are confirmed by other recent studies indicating that service providers report changes in the severity of domestic violence and in perpetrators’ coercive control tactics during the pandemic (Pentaraki & Speake, 2020; Peterman & O’Donnell, 2020; Wood, Baumler, et al., 2020a).

Those shelters that indicated that they thought the COVID-19 crisis and/or infection control measures were being used as a controlling strategy could elaborate on their answers via follow-up questions and free text commentaries. The main categories of coronavirus-related violence observed by the shelters can be defined as forms of coercive control (Stark, 2009, 2013; Stark & Hester, 2019). According to the assessment of the shelters, some of the perpetrators used the lockdown to increase their control over their partner/family by deciding which infection control rules to observe and which rules to violate, by locking up the victim in the home or creating their own “corona rules” to limit the victim’s contacts and social relations. Around half of the shelters that commented on this issue reported that lockdown and the COVID-19 crisis may have increased perpetrators’ stress levels. Coronavirus-related physical and sexual violence was, however, mentioned only by a few.

The free text comments elaborated on how the pandemic and the lockdown could exacerbate the challenges for victims of domestic violence and for particular groups of victims, as well as reflections on possible causes of the decrease in requests. One comment summarized the worries that were expressed by several shelters:

(…)We are concerned that the isolation will hit both children and vulnerable groups extra hard, because we know from experience that the perpetrator gets more leeway and the threshold of tolerance is low.

Another observation from the commentary field regarding how the corona situation had changed the content in the requests from victims emphasized that *“the quarantine and the isolation makes the situation more demanding*.*”* In the first survey the use of the threat *“I will infect you with the virus”* was brought up in the commentary field as an example of changes in the content of requests and coronavirus-related violence. As a result, we added this question in the second survey. However, only one shelter responded positively, which indicates that the direct use of the virus as a threat may not have been an important misuse of the pandemic situation.

Quarantine rules and infection control measures could also become part of parental disputes over custody and visiting arrangements, as this comment illustrates:

(A) former resident returned to us because [her child’s father] refused to comply with quarantine rules and infection control when he was having contact with his own child. (He) joked that he didn’t follow them and still wasn’t sick. Still, he forcibly imposed visits [with the child].

In this case, the deliberate noncompliance with infection control measures becomes an implicit threat of infecting the child. Since it was most poignantly formulated by Stark (2009), it has been well known in the research on domestic violence and coercive control that abusers use a variety of strategies to control, undermine, and threaten their victims. The different uses of the COVID-19 situation by perpetrators as tools of coercive control and threatening behaviors align with the observation from studies of intimate partner violence that, in an abusive relationship, anything may be used as a resource by the abuser (Bjørnholt & Helseth 2019).

To summarize the main findings, it can be concluded that the Norwegian shelters made an effort to maintain services to those victims of violence who needed to stay at a shelter during the lockdown period and after, but most shelters had to reduce or modify their services, particularly to ambulatory clients, in this period. The majority of shelters reported a decline in the number of requests for assistance during lockdown, which was a source of concern that some specific groups of victims might not be able to access services. The number of requests returned back to normal as the strictest infection control measures were lifted in summer 2020. A third of the shelters reported that they had observed signs of corona-related violence, i.e., that the virus or infection control measures had been used by the perpetrators as part of the violence.

Although the shelters remained operative, the pandemic revealed underlying structural problems in society affecting both victims of domestic violence and the shelters, such as the underfunding and lack of resources for shelters and the unequal effects of the crisis on different groups of victims.

## Limitations

The study has several limitations. Firstly, by focusing only on one service, this study does not offer a full picture of all the services available to victims of domestic violence during the pandemic. It is also important to note that the findings are based on two “snapshots”—we need follow-up studies and longitudinal research over time. We are still in the midst of the pandemic, and thus this article is based on the limited knowledge we have at present. On the whole, the survey was rather short, because we did not want to put pressure on shelter staff in the midst of the pandemic. However, in future studies it is important to try to identify additional factors that may be relevant to explore. For example, it would be important to include questions on staff experiences and the challenges of working during a pandemic. Further, although the response rate was high on both occasions (100% and 80%), fewer chose to answer the optional qualitative follow-up questions. Consequently, the sample size for the qualitative part is small, and the representativeness in relation to the whole sample of the qualitative comments is low.

## Concluding Remarks

This study reveals that all in all, the pandemic has not hitherto significantly impacted Norwegian shelters for victims of domestic violence. Despite the need to adapt to the new situation, the shelters have remained open and attempted to pursue a “business as usual” approach, while adapting to the new circumstances caused by the pandemic. This lack of dramatic effects seems to contradict the general crisis narrative with regard to victims of domestic violence during the COVID-19 pandemic. The explanation must be sought in the robustness of the shelters and their institutionalization within the general welfare sector in society. The shelters are today an integral part of the Norwegian universalist welfare state (Esping-Andersen, 1995), and they are part of, and interlinked with, other agencies and service providers, which together form a comprehensive package of public services to persons exposed to domestic violence. However, a major crisis such as the COVID-19 pandemic reveals shortcomings in inter-agency collaborations, including those between shelters and other relevant services such as housing, employment, health, child welfare, and criminal justice. Such cooperation and coordination are of crucial importance, especially in the re-establishment period—that is, after the resident clients have returned to their local community. The longer the COVID-19 pandemic continues, the greater will the need be for more knowledge about the situation of clients after their stay at a shelter.

However, this relative success story is not without serious flaws, and this study also elucidates how the COVID-19 crisis exposes existing inequities in Norwegian society in terms of deficiencies in access to support resources and general societal recognition of the shelters, and thus of victims of domestic violence, which is noteworthy and important for future policies and practices in this area. In Norway, the welfare state offers shelter services as part of a wide range of publicly funded, universal, and high-quality services. Nevertheless, there is noticeable variation within the country with regard to the financial situation and resources available to the shelters, which affects their ability to handle the pandemic in an optimal way. In line with other parts of the Norwegian welfare state, despite its universality, the result for the clients is still dependent on the efforts of frontline staff, who sometimes go to great lengths to bridge gaps to meet the needs of their clients (Vike, 2018). As one shelter put it: *“That we have managed to deliver good services during the corona pandemic has to do with the willingness of our staff to act, to put own needs aside in order to focus more on how to help those who have been exposed to violence in the best possible way.”* As underlined by Wood, Schrag, et al. (2020b), there is a critical need to pay more attention to the experiences and challenges expressed by the frontline staff providing support to victims of domestic violence during the pandemic.

The shelters’ concern that certain groups of victims, including ethnic minorities and migrants, and children and families in vulnerable life-situations, have not received the help they need during the pandemic is corroborated by other recent observations and studies in Norway (Augusti et al., under review; Diaz et al., 2020; MIRA Center, 2020; Øverli & Gundersen, 2020;). Whether shelters and other services manage to reach the most vulnerable groups exposed to domestic violence during a pandemic, e.g., women with a migrant or low-income background, children affected by violence, and persons with substance abuse, mental health problems, or other special needs, has to be explored in more detail by future research. A major societal crisis such as the COVID-19 pandemic may impact vulnerable groups and minorities especially hard and reinforce existing socioeconomic inequalities and power structures. The study indicates that the crisis has highlighted such underlying weaknesses in terms of shelters’ inadequate resources and facilities and the exacerbation of inequalities in the support system for victims of domestic violence in Norway.

As the pandemic and its consequences intensify, its gendered effects begin to attract attention, and this study accentuates the need for more gender-sensitive and intersectional approaches in Norwegian policies on domestic violence in general (Stubberud et al., 2018), as well as for tackling the consequences of COVID-19. Bjørnholt and Hjemdal (2018) highlight how a gender-neutral approach may gloss over persisting gender differences in risks and patterns of domestic violence. Universalist approaches more generally also risk excluding the most marginalized groups, as Richie (2012) has argued. The lack of concern for groups who are disadvantaged based on gender, ethnicity, class, and other social divisions, and the intersection of social structures of marginalization and exclusion, may have important consequences in a crisis.

The present study of Norwegian shelters during the pandemic may be seen as a contribution to research on how services for victims of violence in the pandemic have been resourced and how the services have responded to the present crisis. Identifying and analyzing experiences and responses from service providers and other helpers who are on the frontline can help to prepare for, and strengthen responses to, future pandemics and other crises.

## Footnotes

1 We use the term “lockdown,” well aware that there are important differences in the way in which different countries have implemented lockdowns during the pandemic. One major difference is that in Norway, lockdown has so far not included a curfew. In Norway, COVID-19 infection rates and death rates have been amongst the lowest in Europe throughout the pandemic (see, for example, European Centre for Disease Prevention and Control, 2021).

2 The Istanbul Convention, or the Council of Europe Convention on preventing and combating violence against women and domestic violence, came into force on August 1, 2014, and has (2020) been signed by 45 countries and the European Union (Council of Europe, 2020).
